# Structure of plasmonic multi spectral Apta sensor and analyzing of bulk and surface sensitivity

**DOI:** 10.1038/s41598-024-64249-4

**Published:** 2024-06-10

**Authors:** Mahya Parviz, Younes Majd Shokorlou, Hamid Heidarzadeh

**Affiliations:** 1 Department of Electrical Engineering, University of Kurdistan, Sanandaj, Kurdistan Iran; 2 Department of Electrical and Computer Engineering, University of Mohaghegh Ardabili, Ardabil, Iran

**Keywords:** Biosensor, Plasmonic, Aptasensor, Aptamer, E. coli O157:H7, Biomedical engineering, Electrical and electronic engineering, Engineering, Optics and photonics, Optical materials and structures

## Abstract

In this work, a multispectral aptasensor structure, including a sub-layer and two side walls, was presented. The cells are positioned at the down and top of the structure, with the down cells oriented perpendicular to the walls and the top cells aligned parallel to the walls. The validity of the findings was verified by the utilization of a numerical simulation technique known as 3D Finite Difference Time Domain (FDTD). The biosensor under consideration exhibits sensitivities of 1093.7 nm/RIU, 754 nm/RIU, and 707.43 nm/RIU in mode III, mode II, and mode I, respectively. In the majority of instances, the quantity of analyte available is insufficient to coat the surface of the sensor thoroughly. Consequently, in this study, the evaluation of surface sensitivity was undertaken alongside bulk sensitivity. The surface sensitivity of the suggested structure for mode II in the sensor layer, with thicknesses of 10, 20, 30, and 70 nm, is measured to be 25, 78, 344, and 717.636 nm/RIU, respectively. Our design incorporates a unique arrangement of sub-layer and side walls, with cells positioned to maximize interaction with the target analyte. This innovative configuration, combined with Ag for its superior plasmonic properties, enables the detection of E. coli O157 with remarkable sensitivity.

## Introduction

The importance of food safety is growing every day as different foods are distributed around the global market enables individuals to experience a diverse range of culinary offerings, however accompanied by inherent safety hazards. In order to ensure food safety, it is imperative for the food business to provide products of high quality consistently, to reduce health risks. Food safety is something that people all over the world are worried about. Some chemical hazards; environmental pollutants; natural toxins; Illegal additives and biological hazards can cause adverse effects on humans and the environment^1^ as per the report published by the European Food Safety Authority (EFSA), the most common food pathogens such as Salmonella, Escherichia coli, etc. Within the European Union, it has been determined that the responsibility for a significant number of human cases, over 350,000 annually. However, it is estimated that the number of these cases is more than it is^2^. In the United States, there are approximately 9.4 million cases of illness caused by major known foodborne pathogens, resulting in 56,961 hospitalizations, 1,351 deaths, and $14 billion in economic losses annually^3^.

The sensitive and reliable detection of these pathogens can ensure food safety and act as a practical and essential factor in preventing food infections and significantly reducing hospitalizations, making it an integral part of the development of the food industry. This work requires efficient, easy-to-use, selective, and susceptible methods^4^. Conventional methods to ensure food safety, for instance polymerase chain reaction (PCR), gas chromatography, and high-pressure liquid chromatography, despite being accurate and robust due to the need for expert personnel, are expensive and time-consuming but hardly meet the requirements of the food industry^4^. For these reasons, new methods that are affordable, easy, fast, sensitive, and selective analysis are being investigated, such as optical biosensors.

Inexpensive, portable, sensitive, and integrated instruments that can Deliver achievements in a short amount of time and in real-time are in a significant level of need by the food sector, which is seeking to facilitate expeditious analysis worldwide. The European market for analytical tests designed for food safety applications, particularly in pathogen identification, was valued at over $4 billion in 2018. This substantial figure underscores the significant economic significance of this industry^5^. Most of these studies are about optical biosensors^6^.

Biosensors are integrated and analytical devices, which receive a biological response and convert it into an electrical signal. Biosensors have the selectivity characteristics, independence from physical constraints such as pH and temperature, etc.^7^. Biosensors have been used in several applications, such as environmental appraisal, medical diagnostics, and food analysis^8,9^. Biosensors as high-precision analytical devices use biological receptors to record and analyze biological analytes such as enzymes, antibodies, and aptamers^10^. Biosensors are comprised of a biosensing layer that incorporates antibodies, aptamers, or other biomolecules for target identification, alongside an optical transducer that generates a quantifiable signal^11^. One of the optical biosensors is the plasmonic biosensor, which can simplify the process, reduce time and cost compared to traditional microbiological methods ^8^. The field of plasmonic sensors has witnessed notable progress in recent times. These sensors rely on optical phenomena resulting from the interaction between light and conductive interfaces, specifically thin films or nanoparticles with dimensions smaller than the incident wavelength. This development has paved the way for the creation of biosensors characterized by exceptional sensitivity and selectivity^12^. Plasmonic is used in many sensors and filters, such as^13,14^. The grating can support multiple resonant modes, allowing the sensor to operate at different wavelengths. This can enhance the sensitivity and enable simultaneous detection of multiple analytes. For instance, in^15^, a multi-band Metal–Insulator-Metal (MIM) refractive index biosensor based on Ag-air grating and the feasibility of its fabrication has been presented. They have created the appropriate patterns by depositing the layers from bottom to top using a mask, a deep UV stepper, and reactive ion etching (RIE). When a photoresist is placed above the structure, they obtain the final configuration through the lithography method. So, Electron Beam Lithography (EBL) or Photolithography can create the grating pattern.

The most commonly used plasmonic biosensor is surface Plasmon resonance (SPR), which detects biomolecular interactions^16,17^. LSPR is another type of plasmonic biosensor that offers many applications for sensitive plasmonic biosensing^18,19^. In contrast to SPR, the electromagnetic field in LSPR does not propagate and surrounds metal-bound nanoparticles or nanostructures^20^.

Recently, different materials have been used to change the magnetic properties of materials^21^ that can be used in plasmonic sensors. Plasmonic biosensors can be used for food analysis, require small sample volumes, and can be easily miniaturized^22^. Since it is necessary to ensure food safety and make a simple and quick test of food, which plasmonic sensors solve this need, the possibility of development and progress in this field is evident^23^.

One of the most common pathogenic bacteria is Escherichia coli (E. coli O157:H7)^24^. E. coli O157:H7 is responsible for inducing severe human illnesses, including hemorrhagic diarrhea and hemolytic uremic syndrome, even at deficient infection levels. The fatality rate associated with these disorders ranges from 5 to 10%^25^.

One of the most critical biosensors is their detection part, the most valuable and attractive phage receptor binding proteins and aptamers, which are utilized due to their ability to bind to entire bacterial cells selectively, hence facilitating the streamlining of food analysis procedures^26,27^. Aptamers refer to single-stranded nucleic acid molecules that can to engage with a wide range of targets selectively. Aptamers can increase lifetime and bioavailability through chemical derivatization^28^. Compared to antibodies, aptamers are less expensive, more stable, more accessible to modify, and have fewer or no batch-to-batch variations^29^. Plasmonic aptasensors are devices used in the food industry for the high specificity and sensitive detection of bacteria, such as the detection of S. enteritidis in chicken and fish^30^. Sensitive detection of E. coli O157:H7 using aptamer in ground beef samples^31^.

In many cases, the use of a multispectral sensor is necessary to achieve accurate characterization of biochemical molecules^32^. The ability to monitor at multiple wavelengths is advantageous because it allows for a more detailed investigation of the conformational dynamics and changes occurring in these molecules. For example, multispectral plasmonic biosensors have been suggested as a potential method for detecting prostate-specific antigens^33^. The ability of a multispectral sensor to detect and record the absorption, reflection, or emission of light at different wavelengths offers a plethora of information regarding the presence, concentration, and conversion of specific biochemical molecules, which can be used to discover distinct spectral signatures or Patterns associated with different molecular regions were used and helped to understand their functional roles and interactions in biological systems^34^.

In this research, due to the importance of food health and the need for fast, cheap, and highly accurate diagnosis, we have introduced an aptasensor. In this paper, we investigated the detection of E. coli O157:H7. We are considering, that the proposed sensor is a multi-spectral sensor, architecture comprises a sub-layer and two lateral walls. The cells are positioned at both the down and top sections, with the down cells oriented perpendicularly to the walls, while the top cells are aligned parallel to the walls. Here, a comprehensive analysis of a novel multi-spectral aptasensor structure is designed to enhance both bulk and surface sensitivity for detecting E. coli O157. We propose a unique configuration of a sub-layer and two side walls, with cells strategically positioned to optimize interaction with the target analyte. This innovative design maximizes sensitivity and specificity in detecting E. coli O157. Furthermore, the detailed analysis of bulk and surface sensitivity provides a comprehensive understanding of the sensor’s performance, addressing a critical challenge in biosensing applications where analyte quantities are often limited.

We have investigated the proposed structure, which exhibits many plasmonic modes. We have used FDTD to validate the proposed sensor The results show a multispectral high-sensitivity sensor that can be used in the food industry to detect pathogens in food to improve food safety and health.

## Material and methods

**Figure 1:**
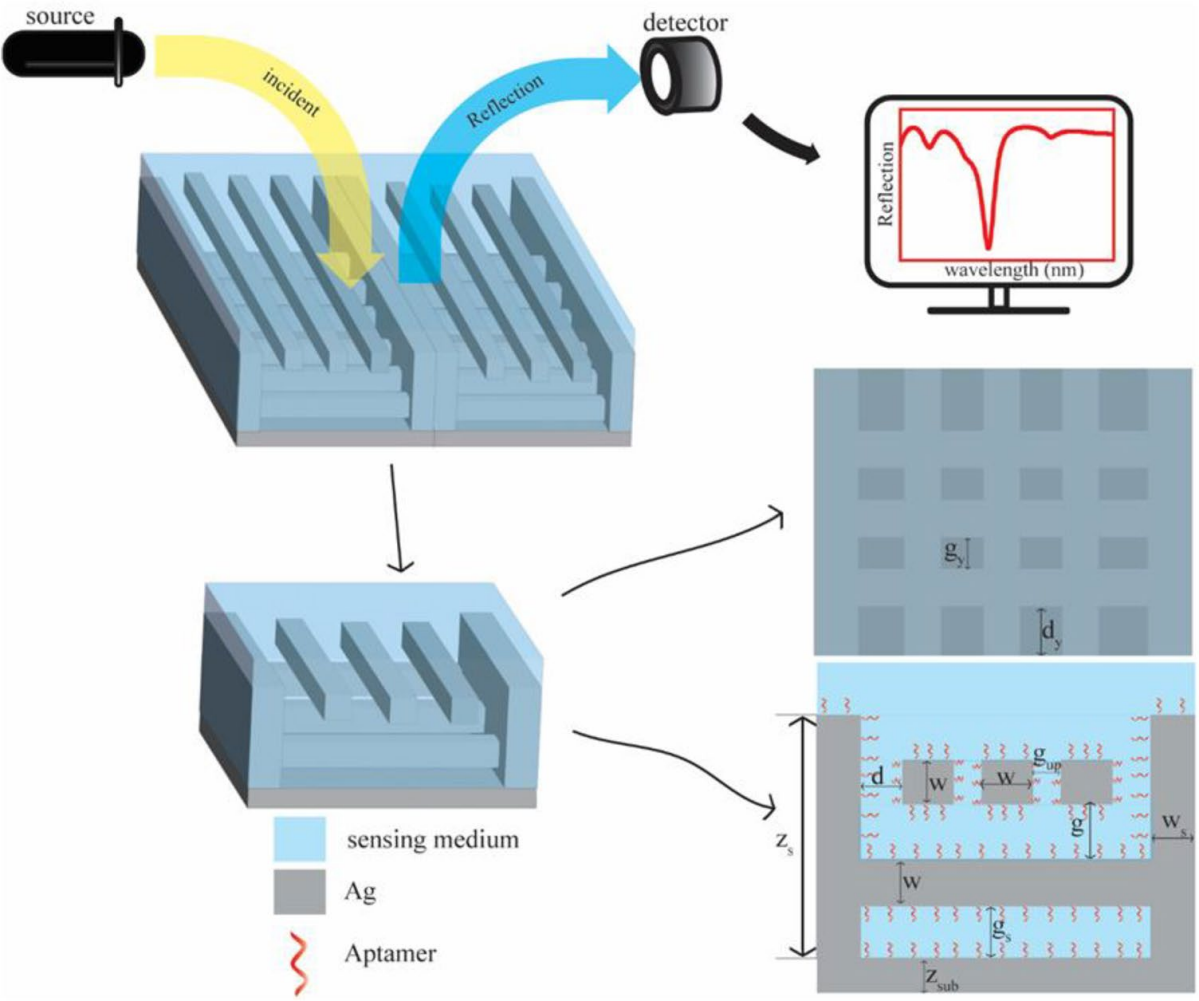
The schematic of the proposed plasmonic aptasensor and a cross-section of a unit cell of the proposed structure are drawn from above and from the side.

In Fig. 1, the proposed structure is shown, which includes a sub-layer and two side walls, and cells are located at the down and top; the down cells are perpendicular to the walls, and the top cells are parallel to the walls. Figure 1 shows a unit cell of the proposed structure. Its dimensional parameters are shown in a cross-section from above and the side. As indicated in the figure, ${z}_{s}$ is the height of the walls, w is the length and width of the cells, d is the distance between the top cells next to the walls, ${g}_{up}$ is the distance between the top cells, g is the distance between the top cells and the down cells, ${w}_{s}$ is the thickness of the walls, ${g}_{s}$ is the distance between the down cells with sub-layer, ${z}_{sub}$ the height of the sub-layer, ${g}_{y}$ the distance of the down cells and ${d}_{y}$ half the distance of the downside cells with the down cells of the next iteration. Optimal values are selected for all parameters using simulation.

The refractive index of the metal is obtained by fitting the experimental data from the handbook of solid optical constant of Edward D. Palik's. in fitting there is the multi-wavelength challenges and some simulators employ combining two or more distinct Lorentz models. To overcome these limitations, Lumerical has pioneered the introduction of multi-coefficient materials (MCMs). MCMs rely on a more extensive set of essential functions to better fit dispersion profiles that are not easily described by Drude, Debye, and Lorentz materials. MCMs can select automatically and optimally the type and number of terms required to describe their materials accurately. The material of the proposed structure is Ag and we have extracted the refractive index information from Palik^35^.

A broadband plane wave light source is employed to excite the plasmonic modes of the aptasensor with wavelengths ranging from 900 to 1700 nm with Transverse Magnetic (TM) polarization. The perfectly matched layer (PML) boundary conditions are used in top and bottom side (the direction of light radiation) and the periodic boundary conditions are applied in other sides (the direction orthogonal to the propagation). The simulation time is long enough to allow the electromagnetic fields to interact with the structure fully.

The measurement performance of plasmonic sensors is based on the changes in the wavelength of the response spectrum, due to the changes in the refractive index of the test medium. The essential main parameters for evaluating the performance of sensors: the sensitivity (S) of Eq. (1), where Δλ is the change in wavelength and Δn is the alteration in refractive index, the full width at half maximum (FWHM), the Q-factor of Eq. (2), which represents the resolution of the detector for specific analytes, where λ is the wavelength of the resonance peak and the figure of merit (FOM) are presented in Eq. (3)^36^.

1 $$ S = \frac{\Delta \lambda }{{\Delta n}} $$

2 $$ Q = \frac{\lambda }{FWHM} $$

3 $$ FOM = \frac{S}{FWHM} $$

The refractive index used for the assay medium is 1.333 for water and 1.388 for E. coli extracted from^37^.

Aptasensor refers to sensors that use aptamers for biological detection^38^. Our proposed aptamer for E. coli detection is used in^39^. Since it is impossible to provide a layer with a specific length and refractive index for aptamers, because they are not identical and there is a space between them, we omitted this layer in our simulation. We performed our simulation without the aptamer layer. There are many methods for building and implementing the aptamer layer, such as the method used in^40^. For the fabrication process of the proposed aptasensor, we suggest the simultaneous use of two methods of electron beam lithography (EBL) in conjunction with thermal evaporation, which is a common practice in the field^41,42^. The reason for choosing Ag for the proposed structure is that it is non-toxic, compatible with biomolecules, and the physical and chemical features exhibited by this substance are remarkable^43^, which leads to the stability of the proposed aptasensor.

Bulk sensitivity and surface sensitivity: Bulk sensitivity considers the change in refractive index for the entire analyte area. Surface sensitivity assumes the change in refractive index in a thin layer close to the surface of the structure^44,45^. Although measurement of Bulk sensitivity is easy and comparable to label-free approaches serve as an approximation for the sensitivity of biosensors in actual biochemical tests. Surface sensitivity, the change in resonance caused by a change in surface refractive index, is a more accurate measure of biosensor sensitivity^45^.

## Results and discussions

In this part of the article, a comprehensive analysis has been done for the optimal selection of the dimensions of the proposed sensor, as well as the measurement of bulk and surface sensitivity. The reflection spectrum results show that the proposed structure has three resonance modes. The purpose of this research is to choose the optimal mode that can be determined through a comprehensive analysis and evaluation of the simulation outcomes.

**Figure 2:**
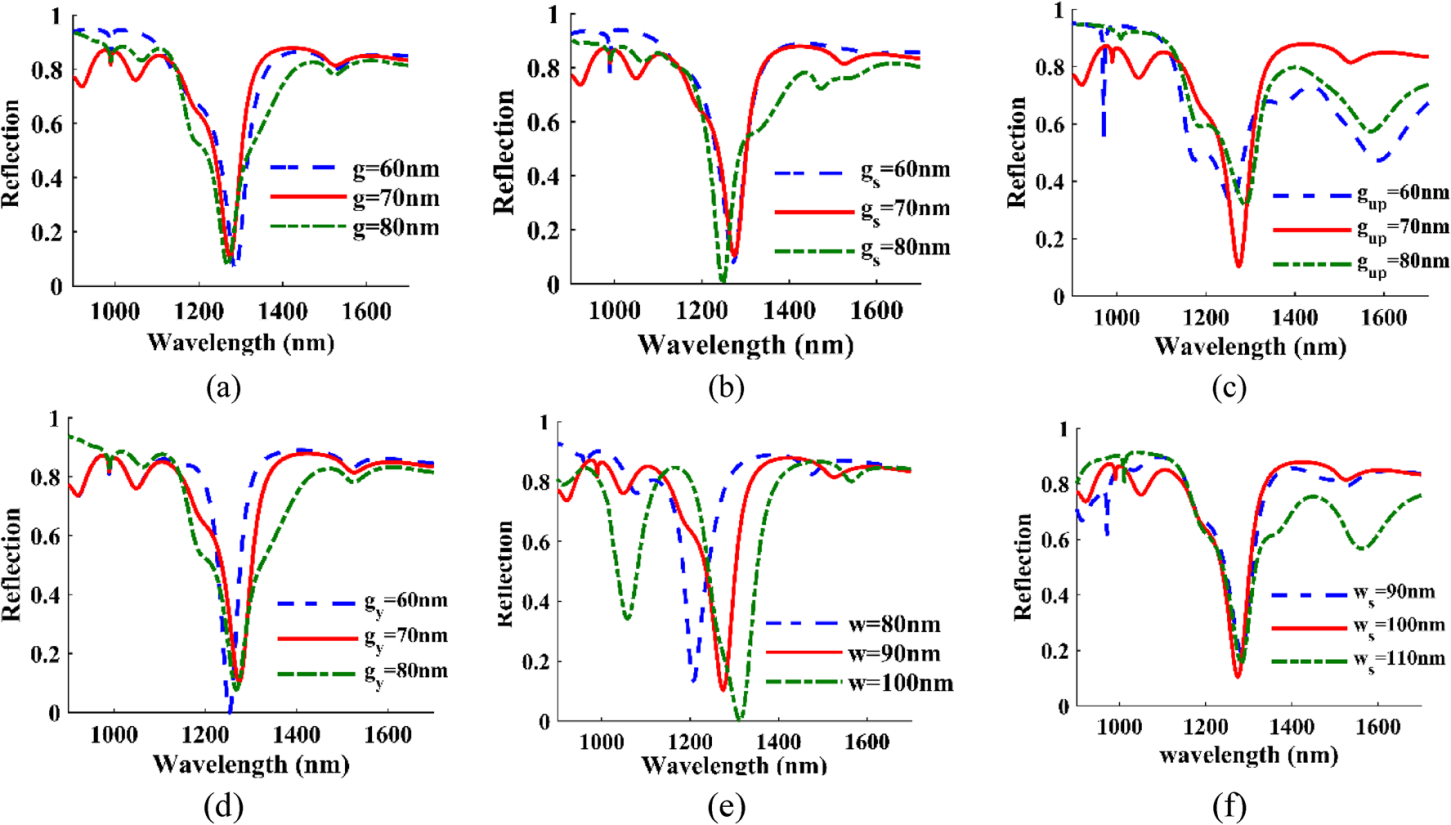
Reflection as a function of wavelength for the different (**a**) distance between the top cells and the down cells g = 60, 70, 80 nm, (**b**) distance between the down cells with sub-layer *gs* = 60, 70, 80 nm, (**c**) the distance between the top cells *gup* = 60, 70, 80 nm, (**d**) distance of the down cells *gy* = 60, 70, 80 nm, (**e**) length and width of the cells w = 80, 90, 100 nm, (**f**) the thickness of the walls *ws* = 90, 100, 110 nm.

In Fig. 2, the effect of the distance between the cells, from each other and the sub-layer, as well as the thickness of the cells and the walls, has been investigated. Based on Fig. 2, the thickness of the cells has a significant effect on the wavelength of occurrence of different modes we can see that the increase in thickness causes a redshift, which has the most significant effect on mode two and the most minor effect on mode one. According to the spectrum of reflection and sensitivity, we choose ${g}_{up}$= g = ${g}_{s}$= ${g}_{y}$= 70 nm, w = 90 nm, and ${w}_{s}$=100 nm.

**Figure 3:**
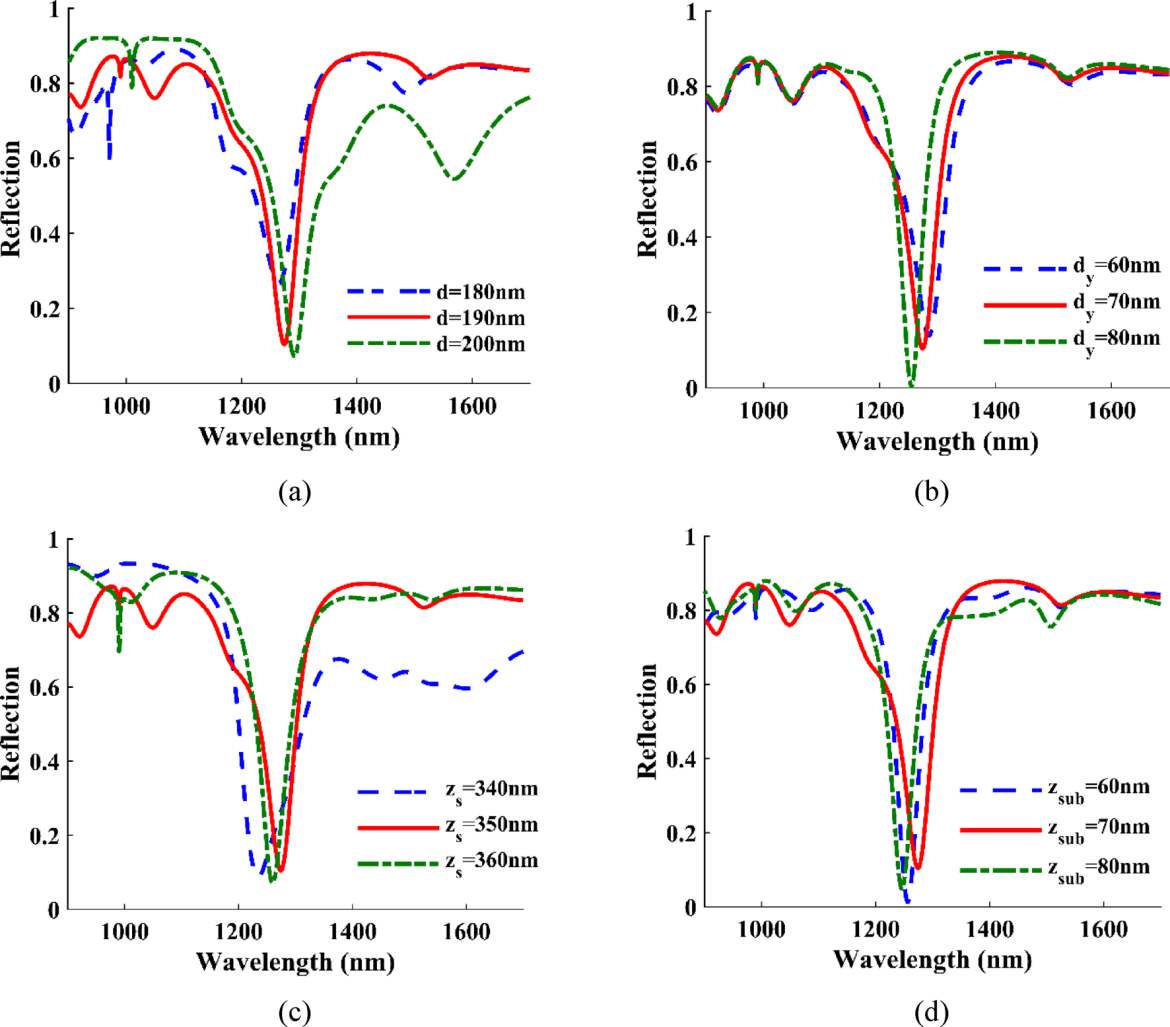
Reflection as a function of wavelength for the different (**a**) the distance between the top cells next to the walls d = 180, 190, 200 nm, (**b**) half the distance of the downside cells with the down cells of the next iteration *dy* = 60, 70, 80 nm, (**c**) the height of the walls *zs* = 340, 350, 360 nm, (**d**) height of the sub-layer *zsub* = 60, 70, 80 nm.

The effect of the distance between the cells and the walls and the height of the sub-layer and the walls are investigated in Fig. 3. For example, ${d}_{y}$ has the most minor effect in modes I and III, and we see a weak blue shift with increasing ${d}_{y}$. Here we choose d = 190 and ${d}_{y}$=70 nm and ${z}_{s}$=350 and ${z}_{sub}$=70 nm.

**Figure 4:**
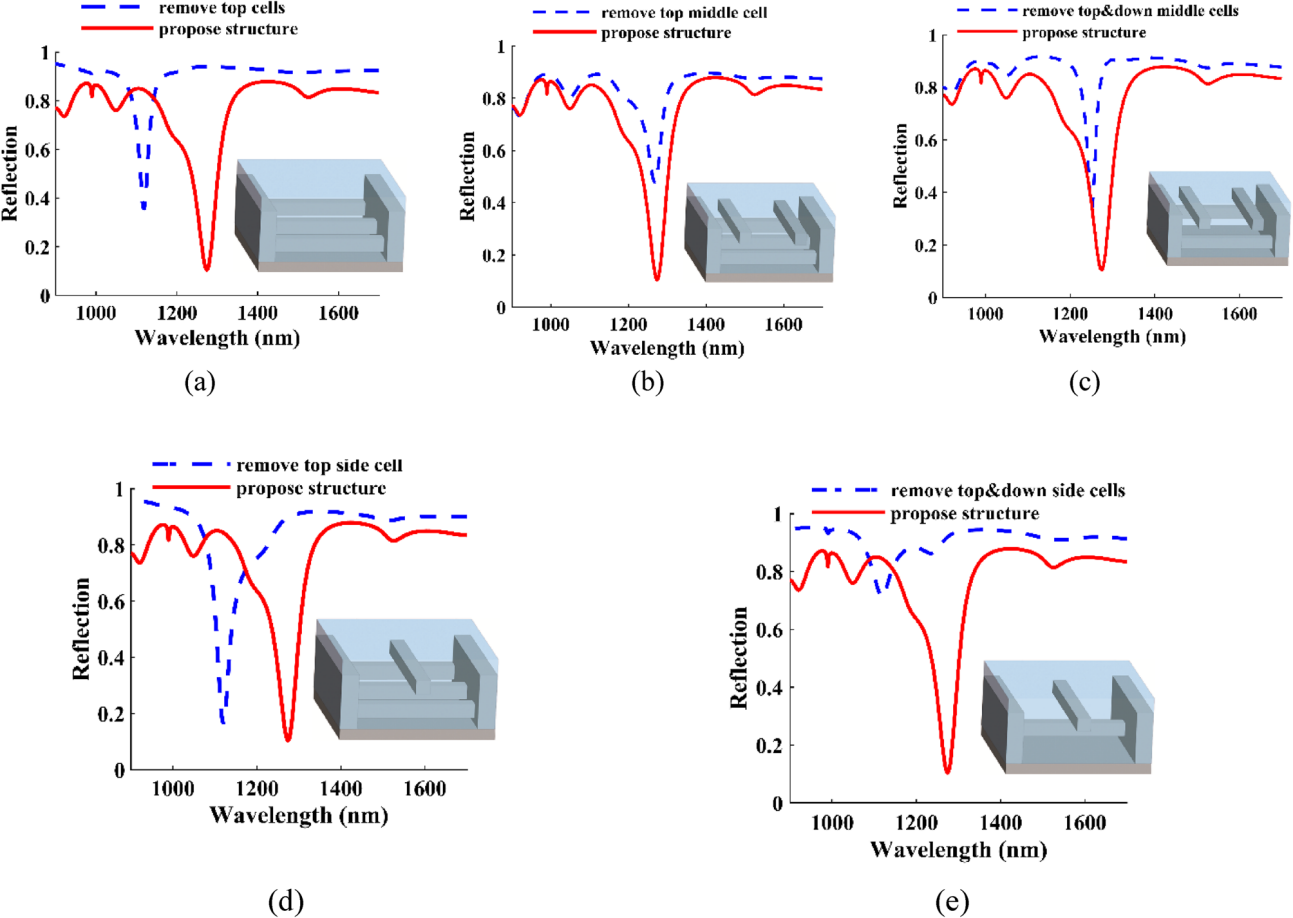
Schematic and reflection as a function of wavelength for the (**a**) remove all the top cells, (**b**) remove the middle cell from the top, (**c**) remove the top and downside cells, (**d**) remove the top side cells, (**e**) remove the top and downside cells.

The effect of cells on the proposed structure can be seen in Fig. 4. In this figure, next to each reflective spectrum, you can see the schematic of the structure. In Fig. 4a, we have removed all the top cells; according to its reflection spectrum, we can see that only one resonant mode is formed. We can see the reflection spectrum of removing the middle cell from the top in Fig. 4b; with its removal, the depth of mode II is reduced, and mode III is almost removed. Removing the middle top and down cells causes a blue shift in the reflection spectrum shown in Fig. 4c. In Fig. 4d, we removed the top side cells, and we can see that this has caused the removal of the first and third peaks and a blue shift in the reflection spectrum of the second peak. By removing the top and downside cells as shown in Fig. 4e, we see that two weak peaks are formed. Considering Fig. 4 and examining it, we conclude that we need all cells to have a reflection spectrum that has all three modes. Upon analysis of Fig. 4, it can be inferred that in order to have a reflection spectrum encompassing all three modes, the inclusion of all cells is necessary.

**Figure 5:**
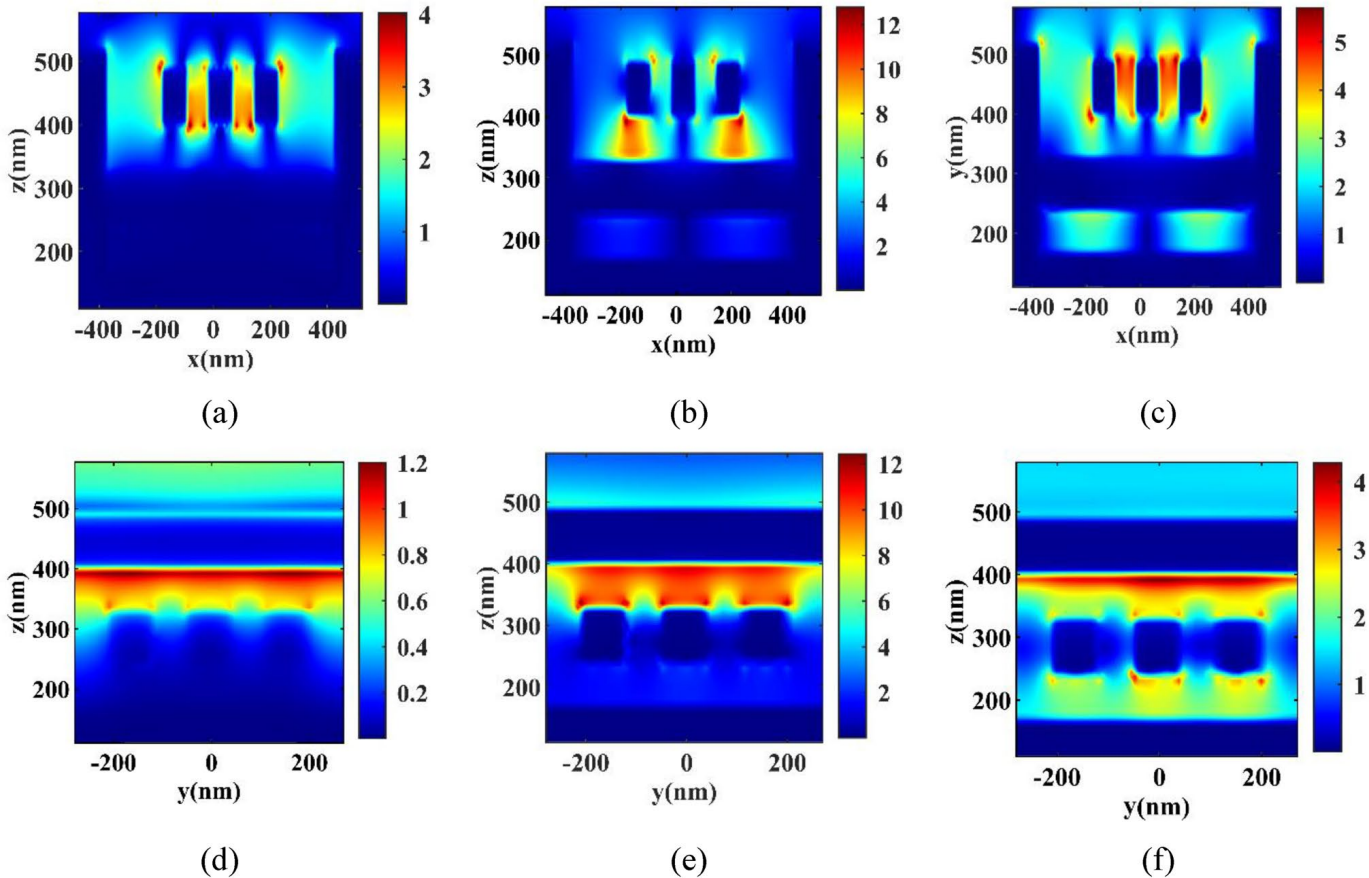
Electric field enhancement from the x and y orientations (**a**) mode I in x orientations at the wavelength of 1048.38 nm, (**b**) mode II in x orientations at the wavelength of 1274.2 nm, (**c**) mode III in x orientations at the wavelength of 1526.56 nm, (**d**) mode I in y orientations at the wavelength of 1048.38 nm, (**e**) mode II in y orientations at the wavelength of 1274.2 nm, and (**f**) mode III in y orientations at the wavelength of 1526.56 nm.

To conduct a comprehensive analysis of the reflection spectrum modes observed in the proposed structure, we present the electric field intensity for all three resonance peaks: mode I at 1048.38 nm, mode II at 1274.2 nm, and mode III at 1526.56 nm. These intensity values are depicted in the x and y orientations, as illustrated in Fig. 5. Based on the observations made in Fig. 5, it is evident that mode II Fig. 5b,e exhibits the highest intensity of the field. In contrast, mode III Fig. 5c,f display a more excellent field dispersion. Additionally, mode III demonstrates the presence of the same field on the sides of the wall, a characteristic not observed in mode II. Due to this rationale, mode III exhibits a higher level of sensitivity than mode II. However, owing to the intensified field strength within the confined region of mode II relative to mode III, more excellent absorption of light is observed. Consequently, this leads to a more pronounced depth in the reflection spectrum of mode II. The mode I of Fig. 5 (a) and 5(d) is less than the other modes in terms of field intensity and field dispersion, which causes relatively lower sensitivity in compared to other modes.

Given the complexity and number of variables involved in the optimization process, it is challenging to present all parameters in detail in a table. Therefore, we have summarized some of the key parameters in Table 1 to provide an overview of the critical factors influencing the sensor's performance. For instance, according above simulated figures it was showed that some parameters affect the resonance conditions, some others influence the strength of the plasmonic resonance and resonance sharpness.

**Table 1:** Summarized results for different parameters in each mode.

Parameter	Dimensions) nm (	Mode	λ (nm)	Sensitivity (nm/RIU)
g	60	I	–	–
		II	1287.1	800.54
		III	1526.56	1119.09
	70	I	1048.38	707.43
		II	1274.2	754
		III	1526.56	1093.7
	80	I	1064.46	727.45
		II	1267.85	777.09
		III	1523.51	1063.63
w	80	I	1079.52	747.81
		II	1207.66	738.90
		III	1476.35	1048
	90	I	1048.38	707.43
		II	1274.2	754
		III	1526.56	1093.7
	100	I	1058.56	645.09
		II	1313.69	795.09
		III	1564.1	1119.81
${w}_{s}$	90	I	1032.77	709.18
		II	1282.77	795.27
		III	1529.62	1123.45
	100	I	1048.38	707.43
		II	1274.2	754
		III	1526.56	1093.7
	110	I	1010.88	708.36
		II	1282.77	795.27
		III	1564.1	1119.81
d	180	I	1032.77	709.18
		II	1265.75	810.36
		III	1484.97	1060
	190	I	1048.38	707.43
		II	1274.2	754
		III	1526.56	1093.7
	200	I	1009.54	709.18
		II	1291.45	805.81
		III	1570.54	1128.90
${z}_{s}$	340	I	950.444	622.45
		II	1235.02	738.18
		III	1600.18	1058.18
	350	I	1048.38	707.43
		II	1274.2	754
		III	1526.56	1093.7
	360	I	1008.21	631.43
		II	1259.48	767.09
		III	1541.98	1141.27

**Figure 6:**
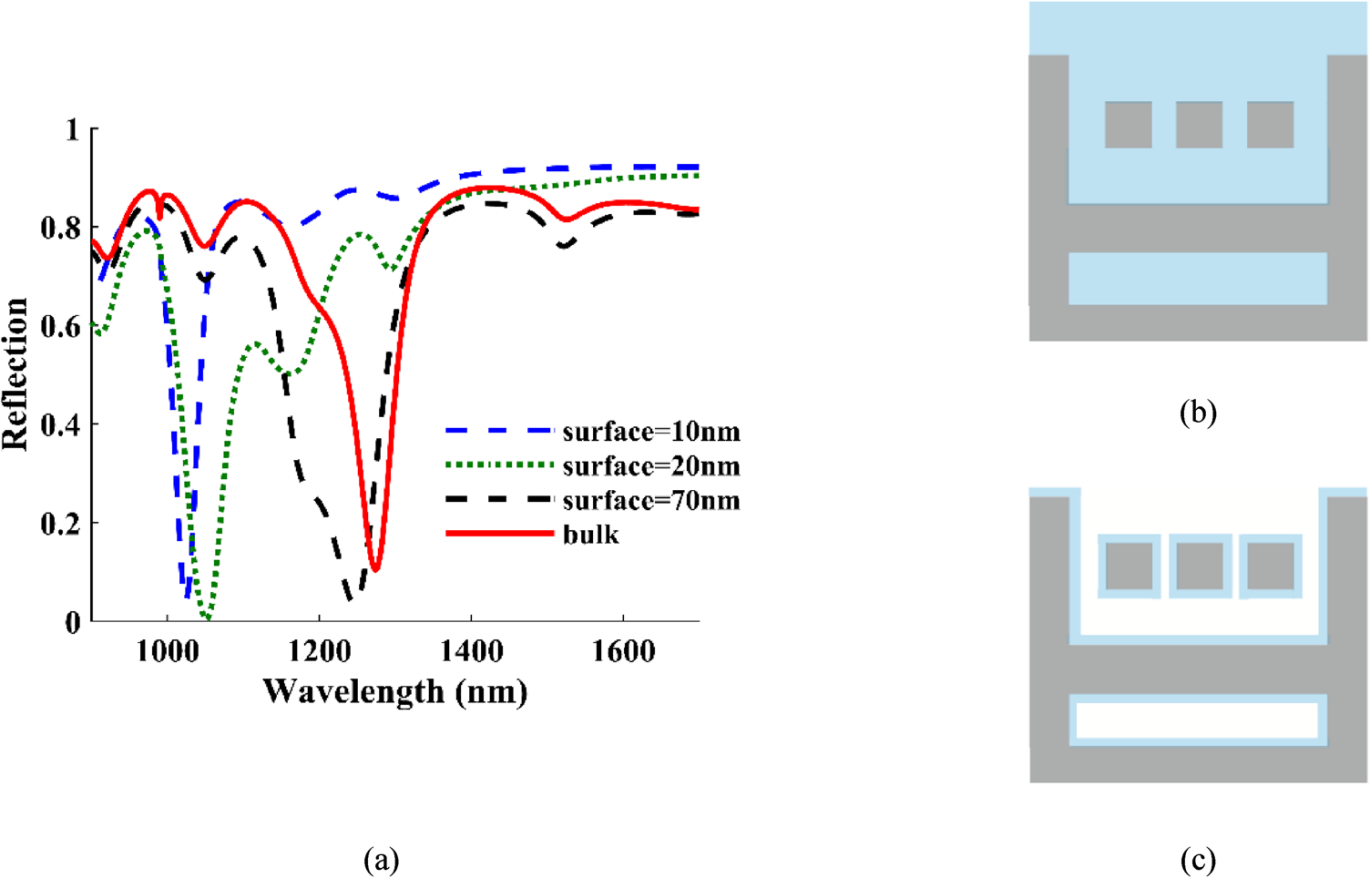
(**a**) Comparison of the reflection spectrum between the suggested structure with a sensor surface layer of 10 nm, 20 nm, and 70 nm, together with the bulk sensitivity and Schematic representation of the (**b**) bulk and (**c**) surface cases.

Detection of food pathogens requires susceptible systems such as plasmonic devices, which in addition to high bulk sensitivity also have high surface sensitivity. Surface sensitivity is predicated on the notion that there is a variation in the refractive index within a tiny layer close to the surface of the structure. Therefore, we considered a thin layer for surface sensitivity analysis. In this section, the surface sensitivity is calculated and compared with the bulk sensitivity. Figure 6 compares the reflection spectrum between the suggested structure with a sensor surface layer of 10 nm, 20 nm, and 70 nm, together with the bulk sensitivity. Additionally, Fig. 6 includes a schematic representation of the surface and bulk sensitivity. In Fig. 6, it can be seen in the reflection spectrum that the increase in the size of the surface layer of the reflection spectrum causes a red shift and approaches the bulk reflection spectrum.

**Figure 7:**
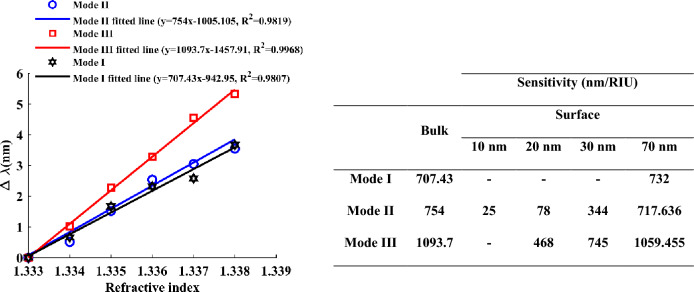
Wavelength shifts as a function of refractive indexes for three different modes of proposed structure and the comparison of surface sensitivity and bulk sensitivity in different modes is presented in the table.

In order to avoid numerical errors and to understand the exact sensitivity of the structure in different modes, as shown in Fig. 7, the change of wavelength as a function of the refractive index is shown. Here, a series of refractive indices is used to derive the average sensitivity for different modes. Also, for each mode, the equation for linear fitting is written as shown in Fig. 7. According to the fitting results, the average sensitivity is 707.43, 754, and 1093.7 nm/RIU for modes I, II, and III. Although the suggested sensor exhibits higher sensitivity in mode III, it demonstrates superior FOM and Q values in mode II compared to the other modes. In the mode II, FOM = 12.03 (${RIU}^{-1}$) and Q = 19.53. The superior FOM and Q values in mode II make it particularly advantageous for applications requiring high precision and accuracy in refractive index measurements. This mode's ability to provide sharper resonance peaks means it can detect small changes in the environment, which is critical for biosensing applications where detecting low concentrations of analytes is required.

Additionally, Fig. 7 presents a comparative analysis of surface sensitivity and bulk across several modes, as depicted in the table. The bulk sensitivity of 754 nm/RIU is obtained for mode II. In contrast, the surface sensitivity for mode II is 25, 78, 344, and 717.636 nm/RIU for the sensing layer with thicknesses of 10, 20, 30, and 70 nm, respectively. It can be seen that with the increase of the layer for the sensitivity of the surface, the sensitivity is close to the bulk sensitivity, just like the reflection spectrum. According to the results, the bulk sensitivity is more than the surface sensitivity, such as ^45^. Aptasensors with high sensitivity are needed to identify pathogens in the low concentration range. Therefore, this proposed structure can be used as a biosensor in cases where high sensitivity is required.

Reliability is paramount for practical sensor applications, particularly in real-world environments. Selectivity, or the ability of the sensor to distinguish between target analytes and interfering substances, is a critical aspect of sensor performance. While our study focuses on detecting E. coli O157, the selectivity of the aptasensor can be further investigated by exploring its response to other bacterial strains or contaminants commonly found in similar environmental samples. Future research could involve functionalizing the sensor surface with specific aptamers targeting different analytes to enhance selectivity. Surface sensitivity is equally essential, especially in scenarios where the quantity of analyte available is limited, leading to incomplete coating of the sensor surface. Our analysis indicates that the surface sensitivity of the aptasensor varies with its thicknesses. Fabrication tolerance-related imperfections refer to deviations from the intended design specifications during the fabrication process. These imperfections can arise from variations in material properties, geometric dimensions, surface roughness, and alignment errors.

In^46^, an aptasensor based on Gold Nanopit Arrays experimentally and numerically has been evaluated and there is a good agreement with these both results. Albumin To benchmark the performance of our multi-spectral aptasensor structure, we compare its sensitivity with recently published works in the field of plasmonic biosensing in Table 2.

**Table 2:** Comparison between this work and sensitivity results of other references.

Reference	Sensitivity(nm/RIU)
^46^	672.36
^47^	562
^48^	450
^49^	541.91
^50^	958.3
proposed structure Mode I	707.43
proposed structure Mode II	754
proposed structure Mode III	1093.7

## Conclusion

In this study, the significance of food pathogen detection is underscored. The research proposes a multispectral aptasensor architecture comprising a sub-layer and two lateral walls. The cells are positioned at both the down and top sections, with the down cells oriented perpendicularly to the walls, while the top cells are aligned parallel to the walls. The proposed aptasensor has three resonance modes to detect E. coli O157:H7. The biosensor under consideration exhibits sensitivities of 1093.7 nm/RIU, 754 nm/RIU, and 707.43 nm/RIU in mode III, mode II, and mode I, respectively. In the majority of instances, the quantity of analyte present is insufficient to coat the surface of the sensor thoroughly. Consequently, alongside evaluating bulk sensitivity, an examination of surface sensitivity was also conducted. The surface sensitivity of the proposed structure for mode II is 25, 78, 344, and 717.636 nm/RIU for the sensor layer with thicknesses of 10, 20, 30, and 70 nm, respectively. The reflectance spectra as benchmarks. A redshift are observed when there is an increase in the refractive index of the surrounding medium. The suggested aptasensor structure has a high bulk and surface sensitivity, making it a promising candidate for biosensors. It can be widely used by changing its aptamer in biological assays and detection of food pathogens. Aptamer-based biosensors have favorable characteristics for assuring food safety. These biosensors possess the advantages of both direct test safety sensors and gene sensors, leading to notable reductions in analysis time and cost. Furthermore, they demonstrate enhanced stability due to temperature and chemical variations. Considering the many advantages of plasmonic biosensors, they also have limitations, such as: reducing non-specific binding to improve performance that requires advances in surface chemistry. The preparation and processing of the test sample are still one of the limiting factors to achieving practical portable biosensors. As a result of the escalating incidence of food-borne illnesses, there is a growing imperative to find novel pathogenic agents present in food. There will be a growing need for devices capable of rapidly and accurately detecting foodborne infections, leading to a projected expansion in the future market. The long-term implications of plasmonic technology are evident, as there is a growing demand for a straightforward and expeditious food testing method that circumvents the necessity of recognized laboratories with intricate licensing prerequisites, warranting future development in the field.

## Data Availability

The datasets used and/or analyzed during the current study are available from the corresponding author on reasonable request.
